# Construction of a ceRNA regulatory network to explore potential pathogenesis mechanisms involved in human hepatocellular carcinoma

**DOI:** 10.1038/s41598-023-47374-4

**Published:** 2023-12-12

**Authors:** Yicun Liu, Zhixing Dong, WeiJie Chen, Lin Chen, Linling Ju, Weihua Cai, Xi Luo, Zhaolian Bian

**Affiliations:** 1 Nantong University Medical School, Nantong, 226001 Jiangsu China; 2 Department of Hepatology Laboratory, Nantong Institute of Liver Disease, Nantong Third People’s Hospital, Affiliated Nantong Hospital 3 of Nantong University, No. 60 Middle Qingnian Road, Nantong, 226001 Jiangsu China; 3 Department of Hepatobiliary Surgery, Nantong Third People’s Hospital, Affiliated Nantong Hospital 3 of Nantong University, No. 60 Middle Qingnian Road, Nantong, 226001 Jiangsu China; 4 Department of Clinical Laboratory, Nantong Third People’s Hospital, Affiliated Nantong Hospital 3 of Nantong University, No. 60 Middle Qingnian Road, Nantong, 226001 Jiangsu China; 5 Department of Gastroenterology and Hepatology, Nantong Third People’s Hospital, Affiliated Nantong Hospital 3 of Nantong University, No. 60 Middle Qingnian Road, Nantong, 226001 Jiangsu China

**Keywords:** Computational biology and bioinformatics, Genetics, Biomarkers

## Abstract

Worldwide, primary liver cancer is the third leading cause of cancer-related death. Hepatocellular carcinoma (HCC) accounts for the majority of primary liver cancers. Recent studies have shown that circular RNAs (circRNAs) that interact with microRNAs (miRNAs) are involved in the occurrence and development of various tumours. Transcriptional profile analysis was used to analyse expression of circRNAs in HCC in this study. The top ten upregulated circRNAs were selected and validated by quantitative reverse transcription polymerase chain reaction (qRT-PCR) in another 34 HCC patients. MiRNAs and mRNAs downstream of these circRNAs were explored through database analysis, and finally, the competitive endogenous RNA (ceRNA) networks were constructed for 5 selected circRNAs. We identified 9658 differentially expressed circRNAs by transcriptional profile analysis. QRT-PCR was performed to validate the top ten upregulated circRNAs, and five circRNAs were selected for further analysis. The miRNAs and mRNAs downstream of these five circRNAs were predicted to construct ceRNA network diagrams. Further analysis revealed five circRNA–miRNA–mRNA axes that correlate negatively with HCC prognosis. Numerous differentially expressed circRNAs exist in HCC, and they can regulate the biological behaviour of HCC through ceRNA networks. Bioinformatics analysis showed that ceRNA regulatory axes involved in HCC have high diagnostic and prognostic value and deserve further exploration.

## Introduction

According to the statistics of world cancer data in 2020, primary hepatic carcinoma (PHC) is the sixth most common cancer in the world and the third cause of cancer-related death, with approximately 906,000 new cases every year, resulting in 830,000 deaths^1^. Compared with statistical results in 2018, the incidence rate and case fatality rate of PHC have shown a gradual increase^2^. There are three types of PHC: HCC, intrahepatic cholangiocarcinoma (ICC), and mixed type. HCC accounts for the highest proportion (75–85%), followed by ICC (10–15%). There are significant differences in pathophysiological mechanism, biological behaviour, treatment regimen and prognosis among the three types of PHC^1,3^. In addition, the prevalence of HCC varies greatly geographically, with the majority of cases occurring in Asia. Many patients with liver cancer have no obvious specific symptoms in early stages, and liver cancer is often discovered incidentally during liver disease follow-up or when combined with liver ultrasound and AFP testing^4^. When symptoms appear, the liver cancer is most often at an advanced stage, and the opportunity for radical surgical treatment is lost. There are several treatment options for patients with advanced disease, but the tumour metastasis and recurrence rates are high^5^. Hence, there is an urgent need to explore new biomarkers to diagnose HCC early and improve the prognosis of these patients.

Recent advances in high-throughput sequencing technology and bioinformatics tools have led to an increasing number of noncoding RNAs (ncRNAs) being found to play an important role in human biological process, especially in the occurrence and development of cancer^6^. NcRNAs include microRNAs (miRNAs), long noncoding RNAs (lncRNAs), and circRNAs^7^. Among these ncRNAs, miRNAs function in post-transcriptional regulation by binding to miRNA response elements (MREs) in target mRNAs. An increasing number of studies have shown that target genes containing common MREs can compete for the same miRNAs. Therefore, in 2011, Salmena et al. proposed the ceRNA hypothesis^8^, which has attracted widespread attention from researchers and stated that coding and ncRNA molecules with common MREs can compete for miRNA binding at these loci and thus indirectly reciprocally regulate expression by acting as miRNA sponges^9^. CircRNAs are a novel class of noncoding ceRNAs and have a circular structure formed by the covalent closure of precursor messenger RNAs (pre-mRNAs) through backsplicing^10^. CircRNAs have no 5′ cap or 3′ tail and are thus not easily degraded by ribonucleases. In addition, they are stable and highly conserved in tissues and cells. Therefore, circRNAs have been widely studied^11,12^.

**Figure 1:**
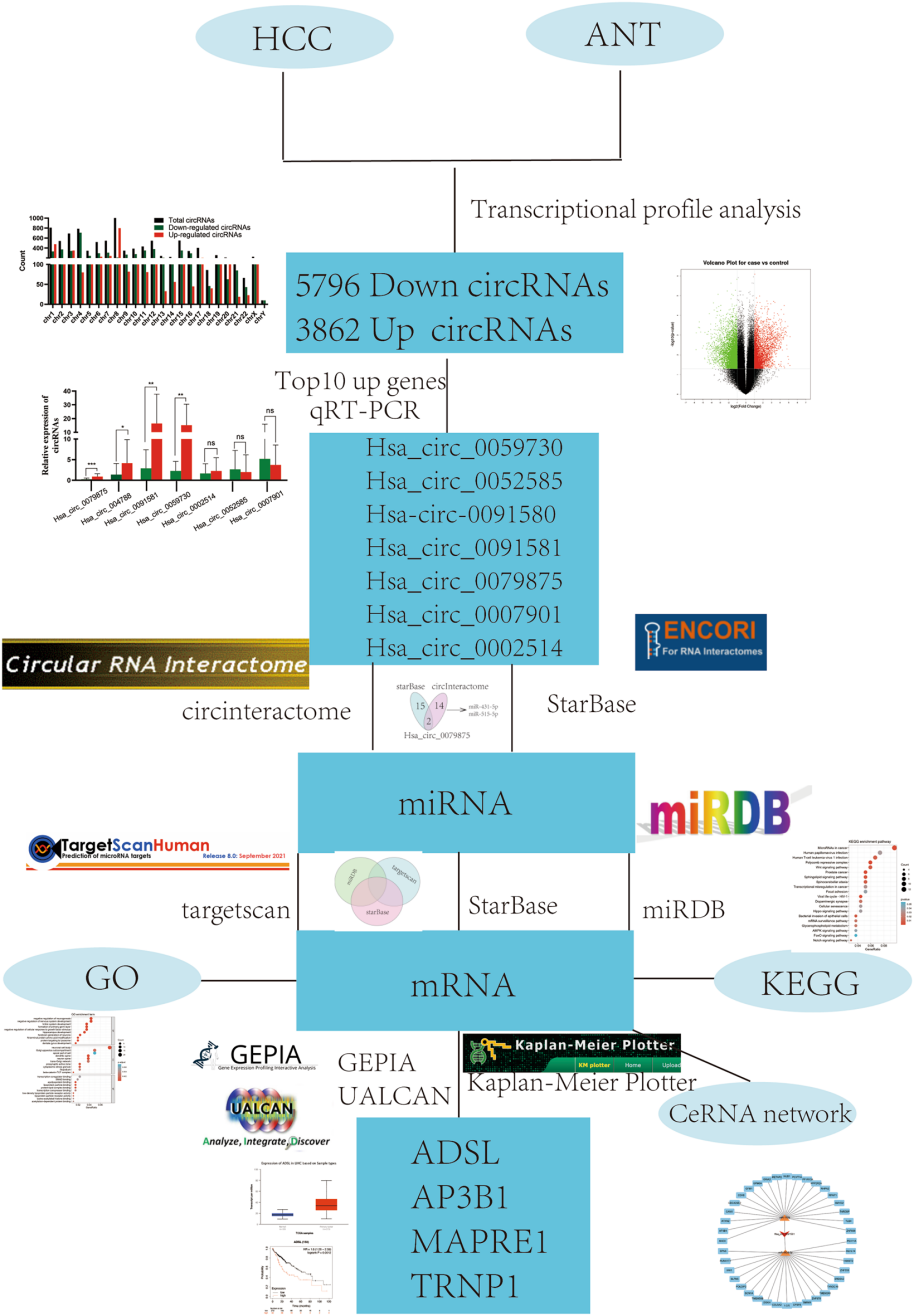
Flow chart of the study. HCC: hepatocellular carcinoma; ANT: adjacent normal tissue; qRT-PCR: quantitative reverse transcription polymerase chain reaction.

In this study, we used a combined strategy of gene microarray analysis and computational biology to investigate novel circRNAs and their potential mechanisms in HCC. A flowchart summarizing the current work is shown in Fig. 1. First, we screened for differentially expressed circRNAs in liver cancer tissues by transcriptional profile analysis and confirmed their expression using qRT‒qPCR. To determine whether circRNAs play roles as ceRNAs in HCC, we identified their sponged and target miRNAs to construct circRNA–miRNA‒mRNA networks. Then, we intersected the mRNAs in the ceRNA networks with upregulated genes in the GEPIA database to verify expression of these mRNAs. Finally, we constructed ceRNA network maps based on ten circRNAs and their respective potential interacting miRNAs/mRNAs to better investigate their potential mechanisms of action in HCC.

## Methods

### Collection of tissue specimens

A total of 34 HCC patients were included in this study. The clinical data of 34 patients are shown in Table 1. Tumour tissue samples and paired adjacent normal tissue samples from these patients were obtained during surgical treatment at the Department of General Surgery, Nantong Third People’s Hospital affiliated with Nantong University, China. The samples were frozened in liquid nitrogen immediately after resection and stored at − 80 °C before use. This study was approved by the Ethics Committee of Nantong Third People’s Hospital, and an informed consent form was signed by every participant. The ethics approval number is EL2021012.

**Table 1:** Clinical data of patients with hepatocellular carcinoma.

Number	Gender	Age (year)	Tumor tissue size (Length*width*height, cm)	TNM
1	Male	57	14*11*8.5	IV
2	Male	58	9.7*9.3*5.7	III
3	Female	56	6.0*5.5*4.0	II
4	Male	36	8.5*6.0*6.0	III
5	Male	65	4.0*3.5*3.5	II
6	Female	49	2.2*2.2*1.5	I
7	Female	57	6.5*5.0*4.5	III
8	Male	35	1.8*1.5*1.2	III
9	Male	55	3.3*2.5*1.6	III
10	Female	62	3.5*3.5*3.0	III
11	Male	38	3.0*1.7*1.6	III
12	Male	51	9.0*6.2*6.0	III
13	Female	68	3.5*3.3*2.7	III
14	Male	55	4.0*2.3*2.0	III
15	Male	69	1.6*1.5*1.5	II
16	Male	66	18*15*8.5	III
17	Male	54	2.9*2.5*2.3	III
18	Male	72	3.5*3.5*3.0	III
19	Male	55	2.0*1.5*1.5	III
20	Male	53	9.0*7.0*7.0	III
21	Female	62	1.5*1.5*1.0	III
22	Male	71	5.5*5.0*3.0	III
23	Female	59	2.0*1.0*0.9	III
24	Male	65	5.5*3.5*3.0	III
25	Male	69	2.0*1.5*1.2	III
26	Male	63	8.5*7.0*7.0	III
27	Male	51	3.5*3.5*2.6	III
28	Male	72	6.7*5.5*5.0	III
29	Female	61	3.5*3.0*2.8	II
30	Male	56	3.9*3.6*2.7	III
31	Male	47	4.0*3.6*2.5	III
32	Male	57	2.6*2.4*1.9	III
33	Male	64	3.5*3.2*3.0	III
34	Male	62	3.5*3.0*2.0	III

### Transcriptional profile analysis

We commissioned CapitalBio Technology (Beijing, China) to perform transcriptional profile analysis. The specific methods were as follows: CapitalBio Technology human circular RNA chip v2 expression profile chip was used to detect the total RNA of samples. In vitro amplification and fluorescence labelling were performed using an Ambion WT Expression kit.

### CircRNA data analysis

The CapitalBio Human CircRNA Array v2.1 was designed with 4 identical arrays per slide  (4 × 180 K format), detects human gene expression, using AG-GE-WL11-01-2010 and AG-GEDL00-01-2010. Post-transcriptional profile analysis was performed by CapitalBio Technology with the following steps. The image after hybridization scanning were preprocessed and converted into quantifiable values by Feature Extraction software. The output of this process is raw data file,which was imported into Gene Spring software, and the grouping and other parameter information were recorded. The “Percentile 75” method of GeneSpring GX software was used for normalization (the ratio was obtained by dividing the 75th digit value of each chip’s own expression data). The value generated after log2 was taken, comments were added, and the data were finally gathered into a total data table that contained the raw and normalized signal values for all samples on the chip, along with the corresponding annotated information. Cluster analysis and graphical presentation were performed using Cluster 3.0 software. Based on the grouping information, differential expression comparisons were performed, and differentially expressed genes were obtained.

### Gene ontology (GO) and Kyoto Encyclopedia of Genes and Genomes (KEGG) enrichment analyses

We performed Gene Ontology (GO) and Kyoto Encyclopedia of Genes and Genomes (KEGG) enrichment analysis on 278 differentially expressed target mRNAs. Functional enrichment of DE genes, using gene sets from GO and KEGG, was determined with Fisher’s exact test, as implemented in the clusterProfiler Bioconductor package^13^.

### Validation of candidate circRNAs using quantitative reverse transcription polymerase chain reaction (qRT-PCR)

Total RNA was extracted from the tissue samples using RNAiso Plus (Takara, Beijing, China) according to the manufacturer’s protocol. Total RNA was reverse transcribed complementary DNA (cDNA) using PrimeScript RT Master Mix (Takara), which was used as a template for determining relative expression of circRNAs with TB Green Premix Ex Taq II (Takara), and the results were normalized to GAPDH as a control. The PCR primers used are listed in Table 2.

**Table 2:** Primer sequences of TOP10 circRNA.

Primer	Accession number	Primer sequence (5′–3′)	Ta	Amplicon size
GAPDH-F	NM_002046.7	CAATGACCCCTTCATTGACC	60 °C	264
GAPDH-R		TTGATTTTGGAGGGATCTCG		
hsa_circ_0091581-F	NM_001164617	GACCACCACTAGGCCTTTGAA		695
hsa_circ_0091581-R		CTTGTGGAGTCAGGCTTGGG		
hsa_circ_0079875-F	NM_018685	AGAAACCAAACAGGAGGAACAGG		299
hsa_circ_0079875-R		TTCTGTGGGGTGTGCTACGA		
hsa_circ_0052585-F	NM_001165931	TGCCTGTGAAGCTCATTGGGA		219
hsa_circ_0052585-R		CCAGGTGTTTGAACATCAGGCA		
hsa_circ_0059730-F	NM_012112	ACGGAGAGAACTGGGTGTTCC		720
hsa_circ_0059730-R		TGCAAAGGCTGGTGACTTGG		
hsa_circ_0004788-F	NM_133631	GAAGTAGCCAGCTCCCGTCT		327
hsa_circ_0004788-R		CGGCCTTCAGCTTTGCAGTT		
hsa_circ_0060361-F	NM_030919	GCTCCTACAGAAAGCAGCCAA		907
hsa_circ_0060361-R		GACAAAAACCGGCGTGCAG		
hsa_circ_0007901-F	NM_018407	ACACTACGACATGTGCATTGCC		392
hsa_circ_0007901-R		TGTACGCTCCGTAAGTAGCCA		
hsa_circ_0002514-F	NM_002073	GGATAACCAGACAGTGAAGGGC		1172
hsa_circ_0002514-R		CTGGTTGCTGTGGCGATGTT		
hsa_circ_0001135-F	NM_012112	TCGAGAAATTGCAACAAAGGTGA		1266
hsa_circ_0001135-R		CCGAGGGGGCATCATAGGAA		

### Prediction of circRNA–miRNA‒mRNA networks

CircRNA–miRNA‒mRNA interactions were predicted by commonly used target gene prediction databases, and networks were constructed by Cytoscape_v3.9.1 software. The CircInteractome (https://circinteractome.nia.nih.gov/), miRDB (http://mirdb.org/), starBase (https://starbase.sysu.edu.cn/) and TargetScan (https://www.targetscan.org/vert_80/) databases were used for specific predictions. For each circRNA, we show the overlapping miRNAs in CircInteractome and starBase. The mRNAs of each target miRNA were predicted by determining the overlap among miRDB, starBase and TargetScan. If there were more than 20 overlapping mRNAs, we screened the top 20 mRNAs based on the miRDB database’s Target Score.

### Selected circRNA–miRNA–mRNA axes

We determined the overlap between all the target mRNAs in the ceRNA networks, with the GEPIA (http://gepia.cancer-pku.cn/) database, and obtained 14 upregulated differentially expressed genes in HCC. Then, we further screened the differentially expressed genes through the UALCAN (http://ualcan.path.uab.edu/) and KM Plotter (http://kmplot.com/analysis/) databases and finally obtained 4 highly expressed differentially expressed genes that correlated negatively with survival. A mechanistic diagram of the ceRNAs was constructed according to the circRNA–miRNA–mRNA action mechanisms.

### Statistical analysis

The circRNA–miRNA‒mRNA ceRNA network diagrams were generated by Cytoscape v3.9.1. GraphPad Prism 8.0 and SPSS 25.0 were used for data analysis. All data are expressed as the mean ± standard deviation (SD) values. Statistical significance was defined as *P* < 0.05 (Supplementary information).

### Ethics approval and consent to participate

All patients were informed of the purpose of the study and signed informed consents, and the collection of specimens was approved by the ethics committee of Nantong Third People’s Hospital (EL2021012). All methods were carried out in accordance with relevant guidelines and regulations.

## Results

### Differentially expressed circRNAs in HCC tissues were analysed by transcriptional profile analysis

**Figure 2:**
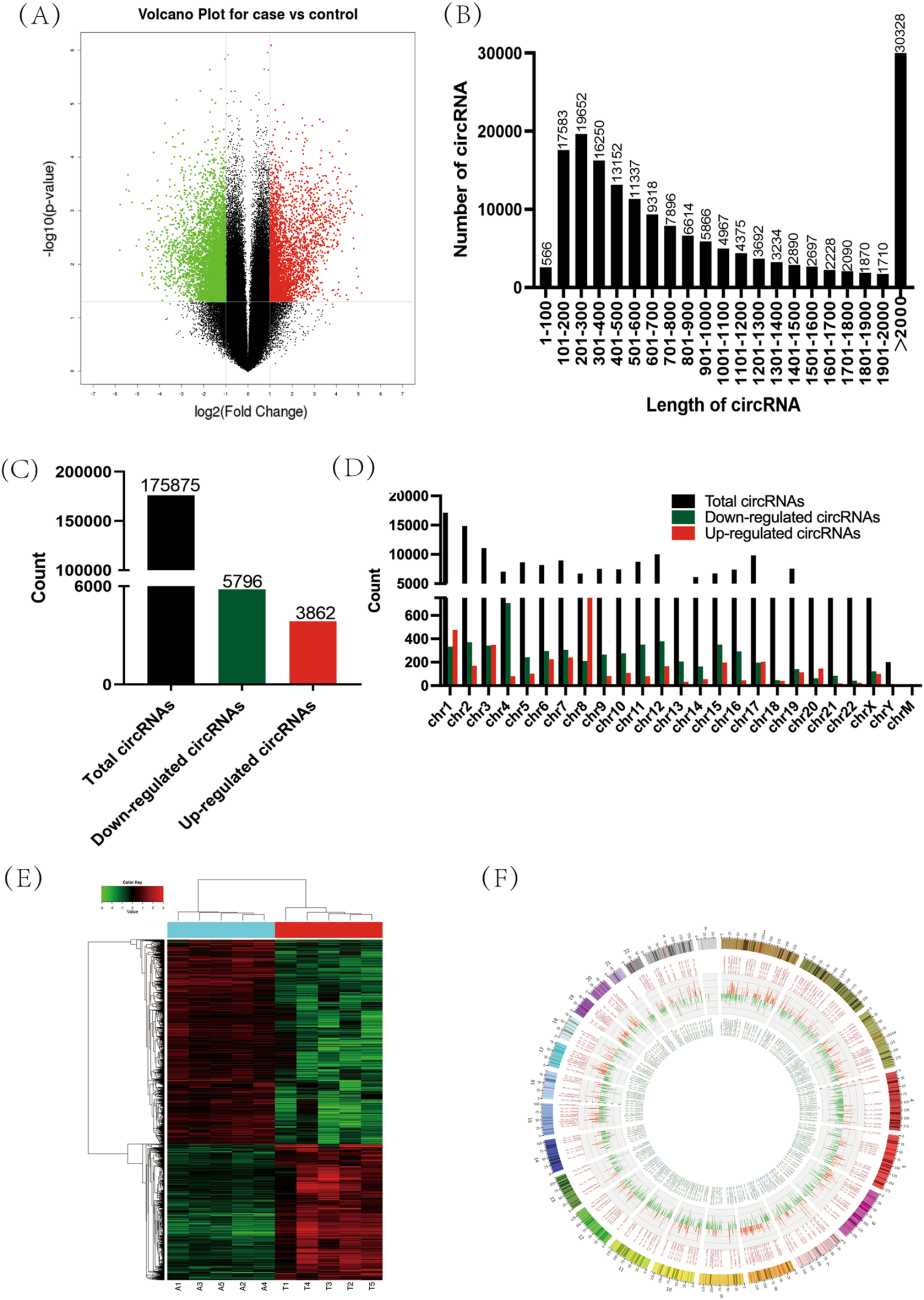
Differentially expressed circRNAs in hepatocellular carcinoma tissues were analysed by transcriptional profile analysis. (**A**) The volcano plot shows the expression profiles in the two groups. The horizontal line represents fold change (log 2). The vertical line represents *P* value (−log10). The red dots in the figure represent the significantly upregulated circRNAs, and the green dots in the figure represent the significantly downregulated circRNAs. (**B**) The length distribution of circRNAs. (**C**) The numbers of total circRNAs and differentially expressed circRNAs. (**D**) The distribution of circRNAs on human chromosomes. (**E**) Hierarchical clustering demonstrated these significant differentially expressed circRNAs. (**F**) The Circos plot clearly shows the degree of differences in gene expression based on the chromosomal location of the genes.

We used transcriptional profile analysis to analyse differentially expressed circRNAs in cancer and noncancerous tissues of five HCC patients. The raw output data of differential expression analysis have uploaded as supplementary information. As shown in a volcano plot, differentially expressed circRNAs were screened with log2 |(fold change)| >1 and *P*<0.05 as the criteria (Fig. 2A). A total of 175,875 circRNAs were identified in tissue samples by transcriptional profile analysis; most of these circRNAs are approximately 100–500 nucleotides in length (Fig. 2B). Among the 175,875 circRNAs, 9658 were significantly differentially expressed: 3862 significantly upregulated and 5796 significantly downregulated (Fig. 2C). In addition, we classified all differentially expressed circRNAs according to their chromosomal locations. The circRNAs on all chromosomes were differentially expressed (Fig. 2D). A heatmap shows these significantly differentially expressed circRNAs (Fig. 2E), and a Circos plot clearly demonstrates the degree of the differentially expressed gene based on chromosomal location (Fig. 2F).

### Validation of differentially expressed genes and prediction of downstream target miRNAs

**Figure 3:**
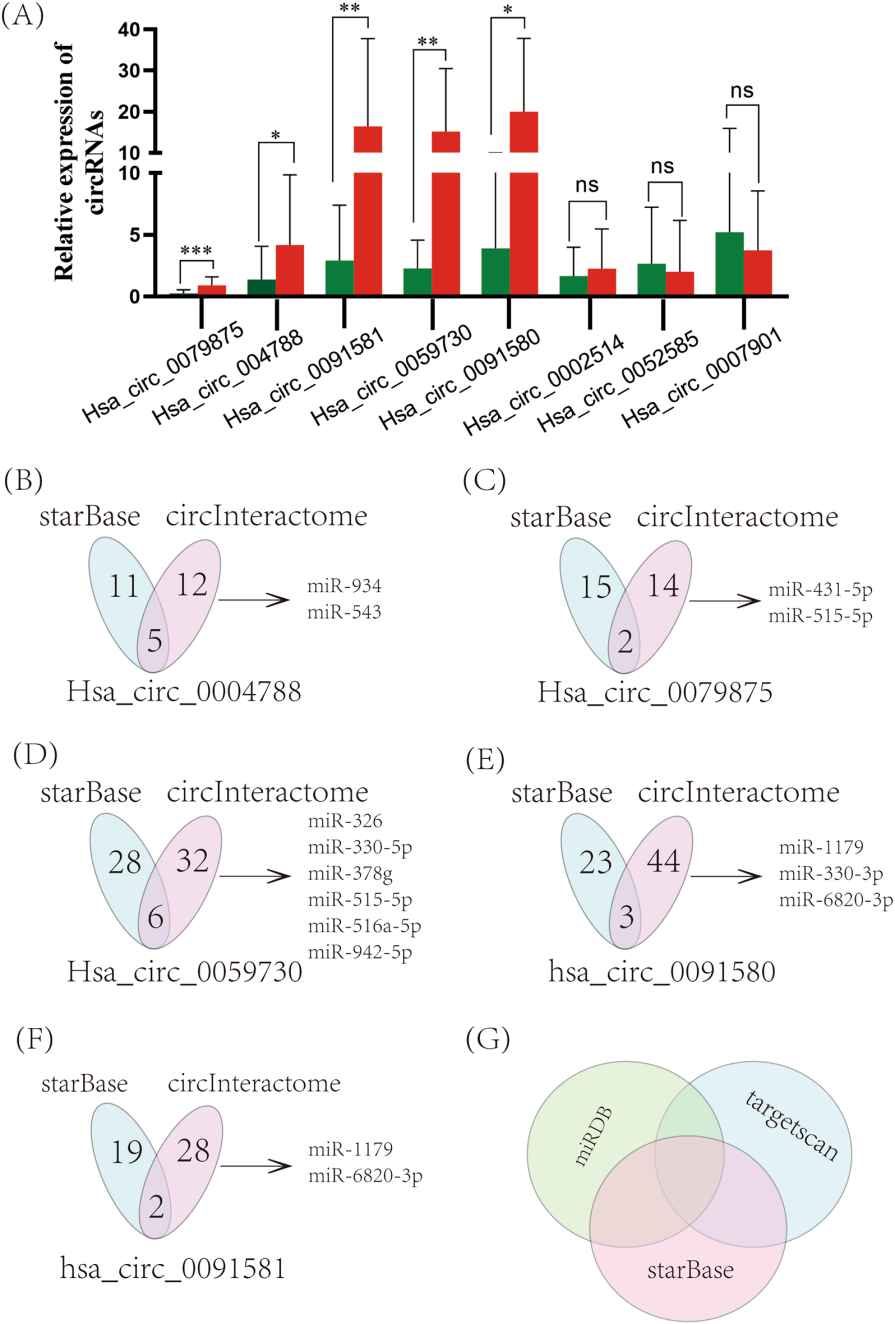
Differential expressed gene validation and downstream target miRNA prediction. (**A**) CircRNA expression was verified by qRT-PCR. The top ten circRNAs with upregulated expression were identified by RNA-seq, and qRT-PCR was used to evaluate their expression in 34 HCC patients, two of which had low RNA expression and are not shown in the figure (**B**, **C**, **D**, **E** and **F**) Prediction of target miRNAs downstream of the five differentially expressed circRNAs (**G**). The mRNA targeted by each target miRNA was predicted by taking the intersection of the results from the miRDB, StarBase and TargetScan databases.

We selected the top 10 circRNAs with the largest difference according to the log_2_FC ranking at *P* < 0.05. To validate the transcriptional profile analysis results of these 10 circRNAs, their expression levels in cancer and noncancerous tissues from 34 HCC patients were verified by qRT-PCR. Hsa_circ_0060361 and hsa_circ_0001135 expression levels were too low to be verified. The results for the remaining 8 circRNAs (6 upregulated and 2 downregulated) were generally consistent with the transcriptional profile analysis results (Fig. 3A). Hsa_circ_0002514 did not show a statistically significant difference because its basic expression was too low. Then, we selected the remaining five statistically significant circRNAs, hsa_circ_0079875, hsa_circ0091580, hsa_circ0091581, hsa_circ0004788 and hsa_circ_0059730, for further analysis. We subsequently analysed these remaining circRNAs with StarBase and CircInteractome (taking the intersection of the results from the two databases to predict the downstream miRNAs of the five circRNAs) (Fig. 3B, C, D, E and F). We used the overlap among three databases, miRDB, TargetScan and StarBase, to predict the downstream mRNAs of these miRNAs (Fig. 3G).

### CeRNA network diagram visualization

**Figure 4:**
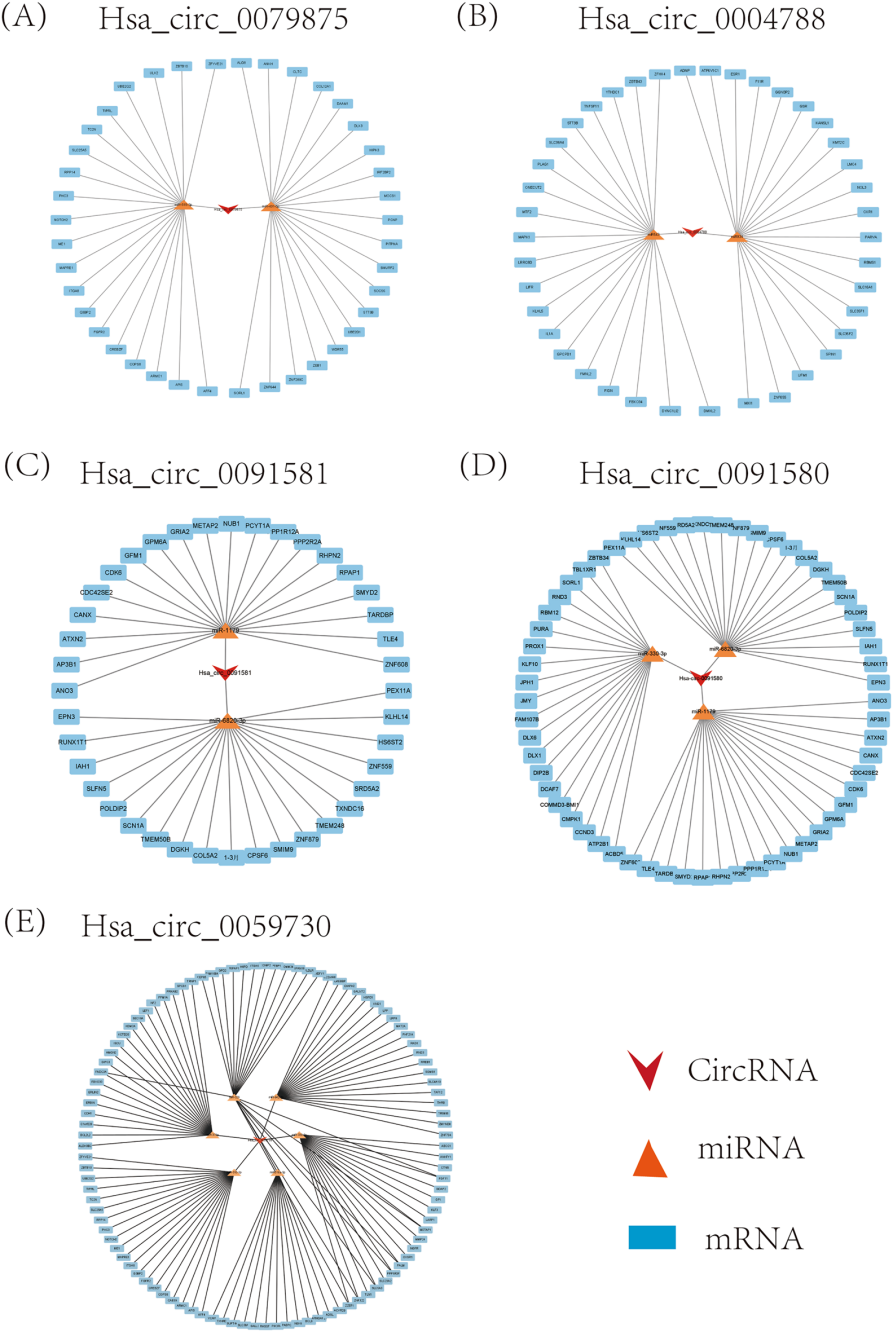
CeRNA network diagram visualization. (**A**, **B**, **C**, **D**, and **E**) circRNA–miRNA‒mRNA ceRNA network diagram.

We predicted the downstream mRNAs of the miRNAs by taking the intersection of the results from miRDB, TargetScan and StarBase. The overlapping mRNAs were screened by the target score in miRDB, and 20 downstream mRNAs of each miRNA were selected to construct ceRNA network diagrams (Fig. 4A, B, C, D and E).

### GO term and KEGG pathway enrichment analyses of mRNAs in the ceRNA network

**Figure 5:**
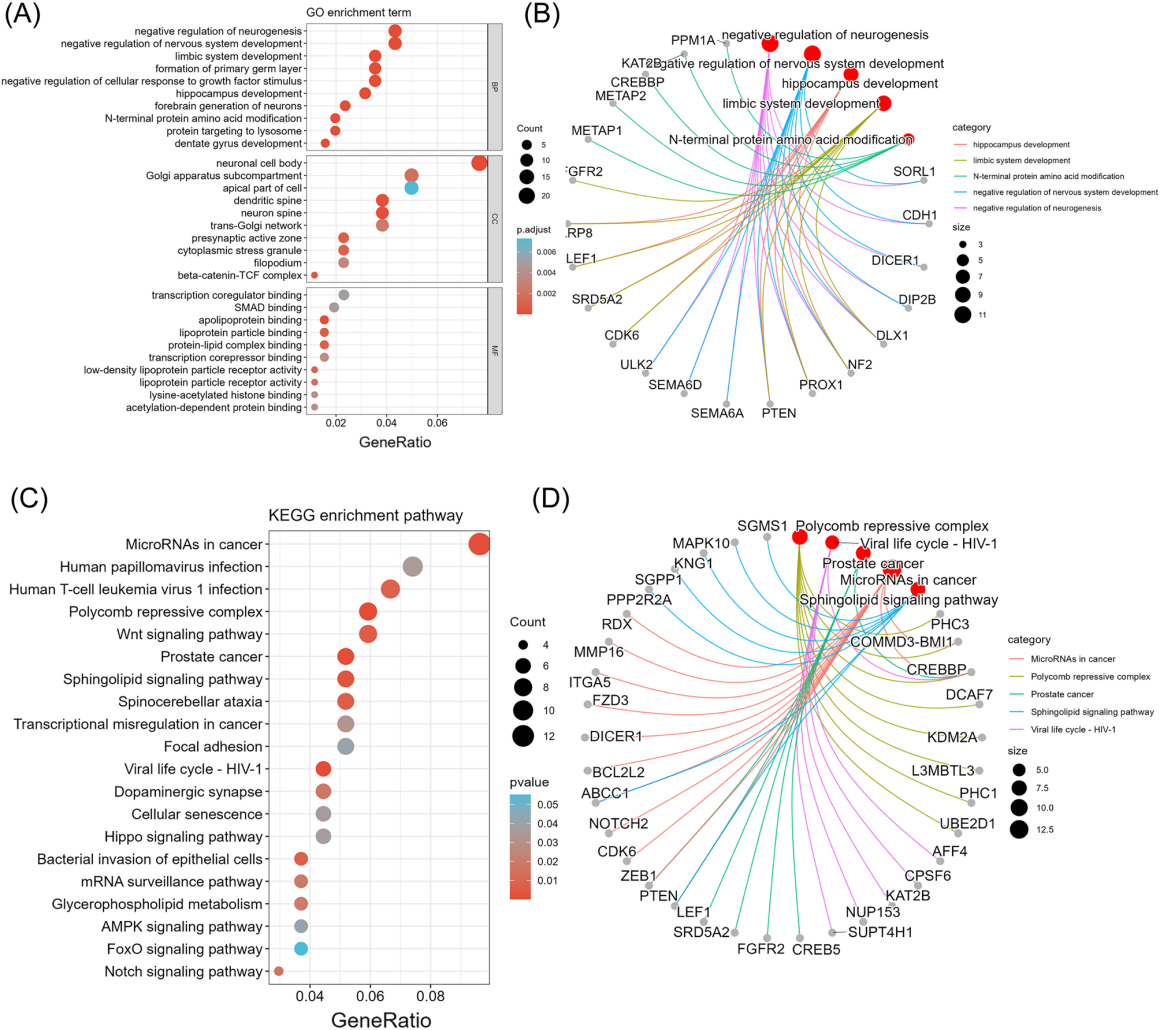
GO term and KEGG pathway enrichment analyses of mRNAs in the ceRNA network. (**A**) GO term enrichment analysis of the mRNAs. (**B**) Top 5 GO networks. (**C**) KEGG pathway enrichment analysis of the mRNAs. (**D**) Top 5 KEGG networks.

GO enrichment analysis includes three categories: cellular component, biological process, and molecular function. In cellular component analysis, the three most enriched terms were neuronal cell body, Golgi apparatus subcompartment and apical part of cell. In biological process analysis, negative regulation of neurogenesis, negative regulation of nervous system development and limbic system development were the three most enriched terms. In molecular function analysis, transcription coregulator binding, SMAD binding and apolipoprotein binding were the most prominent terms (Fig. 5A). We constructed a GO network by intersecting the first five components of the GO enrichment analysis with mRNAs (Fig. 5B). KEGG pathway enrichment analysis showed the functions of the mRNAs in the ceRNA network, microRNAs in cancer, human papillomavirus infection and human T-cell leukaemia virus 1 infection (Fig. 5C). We constructed a KEGG network by intersecting the first five components of KEGG enrichment analysis with mRNA (Fig. 5D).

### Selected circRNA–miRNA–mRNA axes

**Figure 6:**
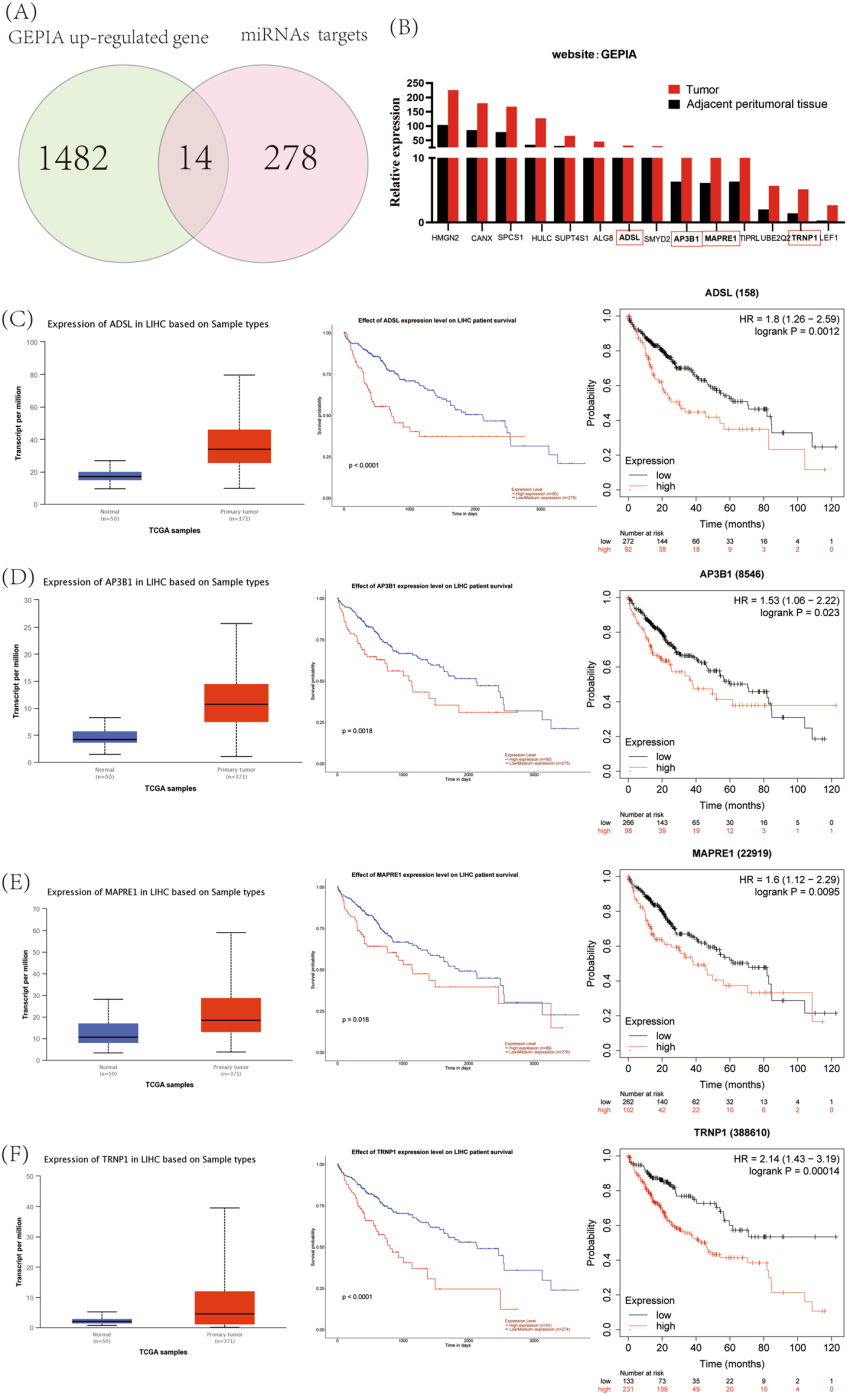
Screening of differentially expressed genes. (**A**) Intersection of upregulated mRNAs with the miRNA-targeted mRNAs in HCC from the GEPIA database. (**B**) Relative expression of 14 upregulated mRNAs in the GEPIA database in HCC and adjacent paracancerous tissues. (**C**) Expression of differentially expressed gene *ADSL* in HCC (UALCAN) and its correlation with prognosis (UALCAN and KM Plotter). (**D**) Expression (UALCAN) and prognostic correlation (UALCAN and KM Plotter) of differentially expressed gene *AP3B1* in HCC. (**E**) Expression (UALCAN) and prognostic correlation of the differentially expressed gene *MAPRE1* in HCC (UALCAN and KM Plotter). (**E**) Expression of the differentially expressed gene *TRNP1* in HCC (UALCAN) and its correlation with prognosis (UALCAN and KM Plotter).

A total of 2207 differentially expressed mRNAs in HCC patients, of which 1482 were upregulated and 725 downregulated, were identified in the GEPIA database. We intersected the upregulated mRNAs in GEPIA with all the mRNAs in our ceRNA network diagrams (Fig. 6A) and obtained 14 candicates. Analysis of relative expression was performed by collating the original data from the GEPIA database (Fig. 6B). Further screening of expression and survival correlations was performed with both the UALCAN and KM Plotter databases, and four differentially expressed genes with high expression and a negative correlation with survival were ultimately obtained: *ADSL, AP3B1*, *MAPRE1*, and *TRNP1* (Fig. 6C, D, E and F).

**Figure 7:**
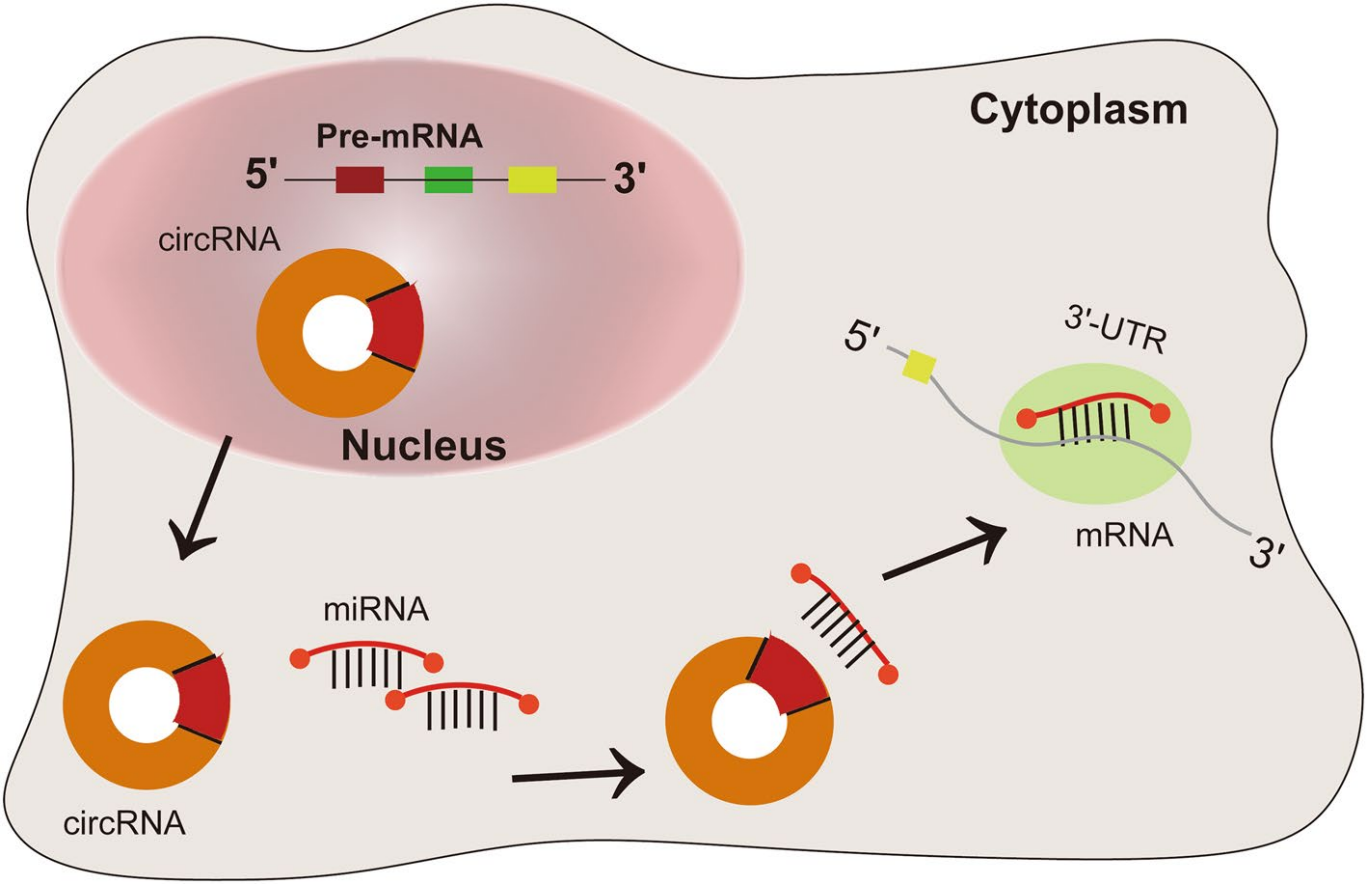
Mechanism of action by which the circRNA–miRNA–mRNA axes affect patient prognosis. The five circRNA–miRNA–mRNA mechanistic axes: hsa_circ_0091581/miR-1179/AP3B1, hsa_circ_0059730/miR-516a-5p/ADSL, hsa_circ_0059730/miR-378g/TRNP1, hsa_circ_0059730/miR-515-5p/MAPRE1, and hsa_circ_0091580/miR-1179/AP3B1.

Based on the above results, we identified 5 circRNA–miRNA–mRNA mechanistic axes: hsa_circ_0091581/miR-1179/AP3B1, hsa_circ_0059730/miR-516a-5p/ADSL, hsa_circ_0059730/miR-378g/TRNP1, hsa_circ_0059730/miR-515-5p/MAPRE1, and hsa_circ_0091580/miR-1179/AP3B1. Finally, we established a schematic diagram based on the roles of the circRNA–miRNA–mRNA axes, as shown in Fig. 7.

## Discussion

Increasing evidence indicates that circRNAs are dysregulated in HCC and can regulate gene expression at the transcriptional or post-transcriptional level, mediating immune escape, drug resistance, proliferation, invasion, metastasis and various malignant biological behaviours of HCC cells^14^. Moreover, circRNAs have high potential as biomarkers for early diagnosis and prognostic assessment^15,16^. These characteristics are verified by the detectability of circRNA in human plasma, saliva, urine and other body fluids^17^. Using transcriptional profile analysis, we found numerous circRNAs (175,875), 9658 of which were dysregulated in HCC and distributed across every human chromosome. Research has confirmed that humans with Down syndrome are at low risk of developing solid tumours due to amplification of several tumour-suppressor genes on human chromosome 21 (HSA21)^18^. Based on this, Peng et al. found that circPTTG1IP derived from HSA21 can inhibit development of HCC^19^. This means that we can infer the possible biological function of circRNA based on the distribution of circRNA chromosomes. Most circRNAs are 100–500 nucleotides long, that is, small molecules. CircRNAs are widely distributed and expressed in all organs and play different roles in various diseases. Many valuable circRNAs deserve exploration.

An increasing number of experiments have demonstrated that circRNAs can promote development of HCC by regulating various signals involved in the cell cycle, proliferation, differentiation, cell survival and apoptosis, leading to abnormal activation of several molecules in signal transduction pathways^20^. Studies have confirmed that circRNAs can act via the AKT/ERK^21^, Wnt/β-catenin^22^, nuclear factor-kappa B^23^, hedgehog^24^, c-Met^25^, JAK2/STAT3^26^ and other signalling pathways and are involved in the malignant biological behaviours of HCC. Recent studies have shown that circRNAs not only exert biological functions through the above signalling pathways but also affect the biological processes of HCC through other pathways. For instance, Liu et al. found that the circRNA cIARS regulates ferroptosis in HCC cells by interacting with the RNA-binding protein ALKBH5^27^, Zhang et al. demonstrated that cancer cell-derived exosomal circUHRF1 induces natural killer cell exhaustion and may cause resistance to anti-PD1 therapy in HCC^28^, and Li et al.^29^ discovered that circRPN2 inhibits aerobic glycolysis and metastasis in HCC. Moreover, Cao et al.^30^ verified that hsa_circ_0003410 promotes HCC progression by increasing the ratio of M2/M1 macrophages through the miR-139-3p/CCL5 axis, Liu et al. found that EIF4A3-induced circTOLLIP promotes progression of HCC via the miR-516a-5p/PBX3/EMT pathway, and Wu et al.^31^ confirmed that methyltransferase-like 3-mediated m6A methylation of hsacirc_0058493 accelerates HCC progression by binding to YTH domain-containing protein 1.

In our study, 5 circRNAs, 15 miRNAs, and 278 mRNAs were identified through transcriptional profile analysis, and 5 ceRNA network maps were constructed of highly expressed circRNAs. A total of 278 mRNAs were further screened, and 4 highly expressed mRNAs with prognostic value were selected. We found that circRNAs can influence the prognosis of patients through ceRNA networks. MAPRE1 promotes cell cycle progression in HCC cells by interacting with CDK2^32^. Adenylosuccinate lyase (ADSL), a key enzyme involved in the de novo purine synthesis pathway, is a potential drug target in HCC^33^. At present, there is no research on AP3B1 and RNP1 in liver cancer, which is worth exploring. In addition, we combined bioinformatics analysis and qRT-PCR results to obtain five circRNA–miRNA–mRNA mechanistic axes that correlate negatively with HCC prognosis (hsa_circ_0091581/miR-1179/AP3B1, hsa_circ_0059730/miR-516a-5p/ADSL, hsa_circ_0059730/miR-378g/TRNP1, hsa_circ_0059730/miR-515-5p/MAPRE1, and hsa_circ_0091580/miR-1179/AP3B1). To date, there has been no research on these five circRNA–miRNA–mRNA mechanistic axes, which are worthy of further exploration. We also performed GO and KEGG enrichment analyses of the 278 mRNAs in the ceRNA network to accurately study the function of these ceRNAs.

Studies on the detailed mechanisms of ceRNA networks and their relationships with HCC are still in their initial stages. Despite increasing interest in exploiting circRNAs as ceRNAs in HCC, little attention has been given to interactions in ceRNA regulatory networks mediated by circRNAs in HCC. Thus, there is a need to investigate the properties, roles and mechanisms of ceRNAs in different stages of HCC. To date, studies of ncRNAs that act as ceRNAs in HCC have mainly involved overexpression and knockdown experiments in cells and animals. However, other factors, such as the subcellular location and abundance of ceRNA components, interactions with RNA-binding proteins, RNA editing, and ceRNA affinity, also affect ceRNA activity. Whether the results of overexpression assays can truly reflect the spontaneous action mechanisms of ceRNAs during carcinogenesis in liver cancer patients remains unknown. Therefore, more animal experiments and clinical trials should be performed for validation. Furthermore, most identified ceRNA interactions show a single binding partner, though ceRNAs crosstalk occurs in large interconnected networks. In addition to direct interactions via shared miRNAs, secondary and indirect interactions may significantly affect ceRNA regulation^34^. Our bioinformatics analysis showed that each circRNA targets several miRNAs; for example, hsa_circ_0091580 targets 64 miRNAs, of which three miRNAs overlapped between two databases. In addition, both hsa_circ_0091580 and hsa_circ_0091581 target miR-1179. According to our study, a circRNA may target several miRNAs at the same time, and we are exploring whether there is a correlation between these miRNAs. Moreover, several circRNAs may also act together on a single miRNA, and whether several circRNAs interact with each other is unknown. Whether there is a correlation between individuals within the ceRNA network will be our next research focus.

However, our study also has some limitations. The results of this study are based on bioinformatic predictions and require further experimental and clinical verification. In addition, data from available datasets are limited and insufficient for prognostic analysis because some circRNAs and miRNAs have not been adequately studied in HCC. We expect that more studies with larger sample sizes will allow us to extend and refine our conclusions.

## Conclusion

In conclusion, by transcriptional profile analysis and integration of data from various databases, we successfully constructed a ceRNA network containing 5 circRNAs, 15 miRNAs, and 278 mRNAs. CeRNA networks contribute to progression of HCC through signal transduction, overall regulation of biological functions, and other pathways. Bioinformatics analysis and qRT-PCR results were combined to obtain five circRNA–miRNA–mRNA mechanistic axes correlating negatively with HCC prognosis, which have high diagnostic and prognostic value and deserve further exploration. Our findings are expected to provide potential biomarkers and therapeutic targets for HCC management.

### Supplementary Information

Supplementary Information.

## Data Availability

All the data are reliable and all the reagents and devices described in the article are commercially available. The transcriptional profile analysis data was uploaded to the GEO database (https://www.ncbi.nlm.nih.gov/geo/query/acc.cgi?) at the NCBI website under accession number GSE242797. We have uploaded the raw output data of differential expression analysis as a supplementary file. The private access token before this manuscript is published is opmpiaugtfivpgf. After the manuscript is approved, we will immediately apply for data publication.

## Abbreviations

circRNAs: Circular RNAs
ceRNAs: Competing endogenous RNAs
HCC: Hepatocellular carcinoma
qRT-PCR: Quantitative reverse transcription polymerase chain reactio
ncRNAs: Noncoding RNA
miRNAs: MicroRNAs
lncRNAs: Long noncoding RNAs
MREs: MiRNA response elements
pre-mRNAs: Precursor messenger RNAs
PKR: Protein kinase R
GO: Gene Ontology
KEGG: Kyoto Encyclopedia of Genes and Genomes
cDNA: Complementary DNA
SD: Standard deviation
HSA21: Human chromosome 21
ADSL: Adenylosuccinate lyase
